# The endoscopic endonasal approach to cranio-cervical junction: the complete panel

**DOI:** 10.11604/pamj.2017.27.277.12220

**Published:** 2017-08-14

**Authors:** Nouman Aldahak, Bertram Richter, Joseph Synèse Bemora, Jeffery Thomas Keller, Sebastien Froelich, Khaled Mohamed Abdel Aziz

**Affiliations:** 1 Department of Neurosurgery, Allegheny General Hospital, Drexel University College of Medicine, 420 East North Avenue, Suite 302, Pittsburgh, PA, 15212, USA; 2 Department of Neurosurgery, Lariboisière Hospital, Assistance Publique, Hôpitaux de Paris, University of Paris VII-Diderot 2, Rue Ambroise Paré 75475 Paris Cedex 10, Paris, France; 3 Departments of Neurosurgery, University of Cincinnati College of Medicine, Cincinnati, Ohio, P.O Box 670515 Cincinnati, Ohio 45267-0515, USA

**Keywords:** Craniocervical junction, endonasal, endoscopic, odontoidectomy, transnasal

## Abstract

We aim to establish a complete summary on the Endoscopic Endonasal Approach (EEA) to Cranio Cervical Junction (CCJ): evolution since first description, criteria to predict the feasibility and limitations, anatomical landmarks, indications and biomechanical evaluation after performing the approach. A comprehensive literature search to identify all available literature published between March 2002 and June 2015, the articles were divided into four categories according to their main purpose: 1- surgical technique, 2- anatomical landmarks and limitations, 3- literature reviews to identify main indications, 4- biomechanical studies. Thereafter, we demonstrate the approach step-by-step, using 1 fresh and 3 silicon injected embalmed cadaveric specimen heads. 61 articles and one poster were identified. The approach was first described on cadaveric study in 2002, and firstly used to perform odontoidectomy in 2005. The main indication is odontoid rheumatoid pannus and basilar invagination. The nasopalatine line (NPL), the superior nostril-hard palate Line (SN-HP), the naso-axial line (NAxL), the rhinopalatine Line (RPL) and other methods were described to predict the anatomical feasibility of the approach. The craniocervical fusion is potentially unnecessary after removal of < 75% of one occipital condyle. A recent cadaveric study stated the possibility of C1-C2 fusion via EEA. This paper reviews all available clinical and anatomical studies on the EEA to CCJ. The approach marked a significant evolution since its first description in 2002. Because of its lesser complications compared to the transoral approach, the EEA became when feasible, the approach of choice to the ventral CCJ.

## Introduction

The transoral approach with its various variations has been the standard route of ventral access for the CCJ [1–5]. This procedure requires a long retraction of the tongue, dissection of the soft palate and incision in the oropharynx below the level of soft palate resulting in morbidity including tracheostomy from tongue swelling, enteral tube feeding or gastrostomy, wound healing complications and velopharyngeal incompetence (hypernasal speech and nasal reflux) due to the incision below the level of the soft palate [6, 7]. In 2002, Alfieri et al [8] published the first anatomical study demonstrated that the CCJ could be sufficiently exposed by the endonasal endoscopic route. This anatomical work gave the start signal to numerous anatomical and clinical studies to develop the Endoscopic Endonasal Approach (EEA). The aim of this study is to identify the evolution of the EEA to CCJ since its first description to achieve a complete summary of surgical technique, anatomical and radiological landmarks that predict the feasibility and limitations of the approach, various indications, biomechanical effect of the approach on stability of CCJ, and finally to demonstrate a step-by-step anatomical study.

## Methods

*Literature review*: we performed a comprehensive literature review of all the English literature published from March 2002 up to June 2015. We utilized the MEDLINE/PubMed database. A bibliographic search was done for further additional articles. No exclusion criteria was considered, all available literature was included including anatomical studies, case reports and patients series. The articles were classified into 4 groups. Group 1- Articles describing surgical techniques; group 2- articles describing anatomical landmarks and limitations of EEA; group 3- clinical patients series and case reports; group 4- biomechanical studies. Each article in each category was reviewed; some articles were included in more than one group.

*Approach demonstration*: one fresh and three silicon injected embalmed cadaveric specimen heads were used to perform the approach for demonstration purpose; a computed tomography (CT) scan was done on the fresh head specimen in order to demonstrate all lines predicting the inferior limit of the approach.

## Current status of knowledge

Sixty-one articles and one poster were identified and reviewed.

*Group 1: articles describing the surgical technique*: Alfieri et al (2002) [8] first described the endoscopic endonasal approach to craniocervical junction in 2002 via an anatomical study utilizing 16 head specimens. Through the endoscopic endonasal route; they were able to expose the whole CCJ from the lower clivus up to the body of C2 without removing the nasal turbinates or the posterior part of nasal septum, this study gave the starting signal for numerous clinical and anatomical studies utilizing the EEA to CCJ. In 2005, Kassam et al [9] was the first to perform a fully endoscopic odontoidectomy in a 73-year-old woman with a history of rheumatoid arthritis and suffering from craniocervical junction ventral compression. Kassam identified his newly described approach among the expanded endoscopic approaches to the anterior skull base. One-side removal of the middle turbinate, a wide sphenoidectomy and removing 1cm of the posterior nasal septum allowed enhancing his surgical exposure. One year later, in 2006, Kassam [10] used his expanded EEA for clipping a large vertebral artery aneurysm.

In 2007 the department of neurosurgery in the University of Naples Federico II/Italy published two anatomical studies demonstrating the feasibility of the EEA to decompress the ventral CCJ and the upper cervical spinal cord [11, 12]. First one performed by Messina et al (2007) [12] who described the possibility of odontoidectomy by the EEA without any turbinectomy or even without the removal of the posterior third of the nasal septum. The second work was done by Cavallo et al (2007) [11] who exposed the entire clivus and CCJ using the expanded approach associated with opening the sphenoid sinus. The first retrospective patients' series was published in 2007 by Nayak et al [13]. Nine patients underwent endonasal endoscopic resection of the odontoid process. Since then, numerous studies have described the approach in adult and pediatric patients; they varied between patients' series, case reports and anatomical studies [14–45]. Technically, in order to access a midline lesion limited to CCJ region, many authors accept the use of two nostrils approach without turbinectomy or sphenoidectomy but with removing the posterior 1-2cm of the nasal septum to enlarge the choana and facilitate binostril application of instrumentations [16, 17, 24, 26, 29, 34]. The removal of anterior floor of the sphenoid sinus is another option to enlarge the working space [9, 10, 30, 36]. However, the use of one nostril or two nostrils technique and/or the expansion of the surgical corridor are strictly related to the lesion encroachment, surgeon's experience and/or preference.

**Figure 1:**
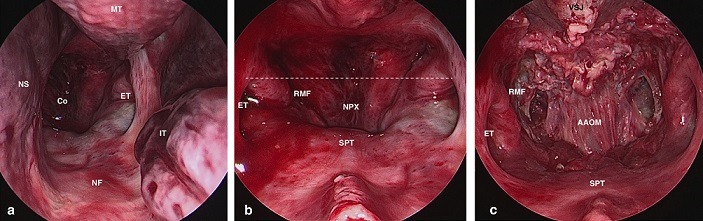
(A) 0° endoscope is introduced in left nostril of fresh head specimen the free end of the middle turbinate leads to the superior lateral aspect of the choana: (Alfieri et al) IT= inferior turbinate; MT= Middle turbinate; NF= Nasal floor; NS = Nasal septum; Co= Choana; ET= Eustachian tube; (B) removal of 1-2 cm of posterior nasal septum, the endoscope is placed on midline. RMF = Rosenmüller fossa; SPT = Soft palate; NPX = Nasopharynx; the line drawn between the upper edges of the eustachian tubes ostia (dashed line) crosses the ventral margin of foramina magnum “landmark” (Alfieri et al 2002); (C) mucosal-muscular flap was done and pushed into mouth cavity VSJ= Spheno-vomer junction; AAOM= Anterior Atlanto-occipital membrane (or ligament)

**Figure 2:**
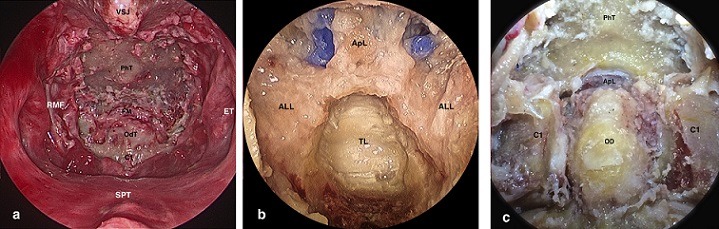
(A) bone exposure after removal of AAOM: PhT = Pharyngeal tubercle of clivus; FM = Foramina magnum; OdT= Odontoid tip; C1 = Anterior arch of C1; (B) embalmed specimen: removal of anterior arch of C1 apL = Apical ligament; OD= Odontoid process; (C) the odontoid process was hollowed out and removed; the clivus was partially drilled in order to well demonstrate the apical ligament (ApL); the C1 superior articular process was also partially drilled on each side to show the Alar ligaments (ALL) TL = transverse portion of cruciate ligament

**Figure 3:**
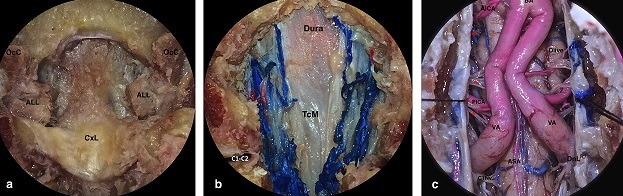
(A) the apical ligament was removed; the alar ligaments were cut out. The C2 body and was partially drilled CxL= Cruciate ligament; Occ = occipital condyle; (B) removal of cruciate ligament TcM= Tectorial membrane; (C) intradural view (sutures were applied on dura by transoral route for demonstration purpose C1Nv = C1 ventral nerve roots; DnL= dentate ligament; ASA = Anterior spinal artery; VA= vertebral artery; PICA= Posterior inferior cerebellar artery; AICA = Anterior inferior cerebellar artery; BA = Basilar artery

**Surgical steps**: A 0° endoscope is introduced in the nostril. The free end of the middle turbinate leads to the superior lateral aspect of the choana [8]. (Figure 1 A); 1-2 cm of posterior nasal septum is removed in order to place the endoscope on midline. The line drawn between the upper edges of the Eustachian tubes Ostia crosses the ventral margin of foramina magnum “landmark” [8]. (Figure 1 B); tarious nasopharyngeal incisions could be used [25]. We demonstrate a mucosal-muscular flap, which will be pushed into mouth cavity (Figure 1 C). Bone exposure: the anterior atlanto-occipital membrane (AAOM) is removed (Figure 2 A); the anterior Arch of C1 is removed; therefore, the odontoid process and ligaments are visualized (Figure 2 B). The odontoid process is hollowed out then removed (Figure 2 C); the cruciate ligament is well exposed by removal the apical ligament and cutting out the alar ligaments (Figure 3 A); the extradural exposure could be done by removal of cruciate ligament if needed (Figure 3 B); the dura is opened for intra dural lesions (Figure 3 C).

*Group 2: feasibility and anatomical limitations of the approach*: the EEA to the CCJ is limited by anatomical structures, superiorly by the bony and soft tissues of the nose and inferiorly by the hard palate. Messina et al (2007) [12] stated that the possibility of performing an odontoidectomy via an endonasal route is strictly related to the level of the C1-C2 junction, which must be above the level of the horizontal hard palate, otherwise this procedure is virtually impossible. Since there, authors are trying to accurately predict the EEA limitations and thus, to choose the suitable approach for each patient without limiting the surgical exposure. The Palatine Line (PL) or the Hard-Palate Line (HPL) which drawn parallel to and along the floor of the hard palate was used as a reference in almost all studies [43, 46–52]. De Almedia et al (2009) [53] reviewed retrospectively the computed tomographic (CT) scans of 17 patients who underwent endonasal endoscopic surgery of the C1 or C2 vertebrae, they defined the nasopalatine line (NPL) connecting the most inferior point on the nasal bone (rhinion) to the most posterior point on the hard palate in the midsagittal plane, this line was called “Kassam Line” later on according to the senior author. De Almedia et al stated that the point of intersection of the NPL and the vertebral column accurately predicts the inferior limit of the surgical exposure.

Baird et al (2009) [54] based on a cadaver study on 9 specimens defined a line from the rhinion to within 3 to 5 mm from the base of the surgical resection. They stated that EEA is feasible as long as this line doesn't cross the posterior end of the hard palate. Aldana et al (2012) [55] presented a poster of 6 cadaver specimens study claiming that the previous described NPL or Kassam line overestimates the inferior limit of EEA to CCA, for that, they presented a new line, the superior nostril-hard palate line (SN-HP Line) that connects the superior aspect of the nostril to the anterior border of C1-C2 junction. Later on, the same author [46], stated that his (SN-HP Line) underestimated the inferior limit of the EEA. He described the naso-axial line (NAxL) where the starting point is the midpoint of the distance from rhinion to the anterior nasal spine of the maxillary bone and a second point is the tip of the posterior nasal spine of palatine bone. The line then extended to end at the spine column. Singh et al (2013) [51] analyzed the CT scan of 100 patients and found an inverse relationship between hard palate length and the lowest zone of the cervical spine potentially visualized by nasal endoscopy, this was the first study where the visualization by a 0, 30 and 45-degree endoscope was simulated. Same authors completed their clinical study by a second anatomical part [52] and concluded that the use of a 30-degree endoscope and neck extension increased the degree of exposure down the cervical spine.

**Figure 4:**
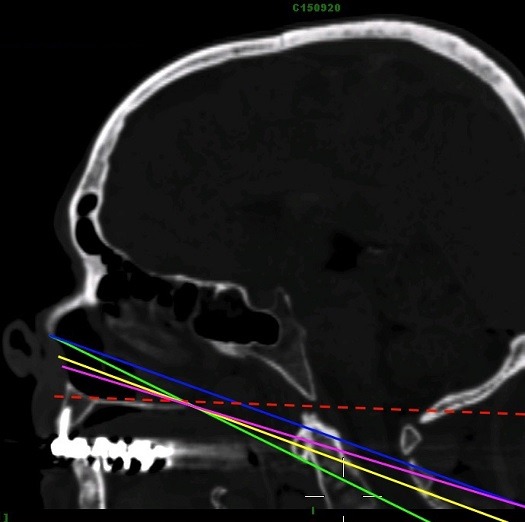
Lines predicting the inferior limits of EEA to CCJ, NPL (green), SN-HP Line (blue), NAxL (yellow), RPL (Purple), the hard palate line (HPL) is also shown in dashed red

Lin et al (2014) [56] stated in their radiological study on 34 patients that there is no influence of the position of the cervical spine on the surgical convenience of the endoscopic transnasal approach. La Corte et al (2015) [50], found that the previous described NaxL overpredicted the approach limits, for that and depending on CT scan performed in patients who underwent an odontoidectomy, they described a new line, the Rhinopalatine Line (RPL) where the starting point corresponds to the two-thirds point of the distance from the rhinion to the anterior nasal spine of the maxillary bone and a second point at the posterior nasal spine of the palatine bone. The line is then extended to end at the cervical spine. Corte et al. stated that their new described line is the most accurate one that predicts the actual inferior limit of EEA to CCJ. The main lines described in literature to predict the inferior limitation of EEA to CCJ are summarized in Table 1 and Figure 4.

**Table 1:** Lines predicting the inferior limit of the EEA to CCJ

Study/Year of publication	Type	Line ^§^	Starting point	End point	Line extension	Comments
De Almedia et al 2009	17 patients	Nasopalatine line (NPL)	Rhinion^*^	Most posterior point on the hard palate	Posteriorly and inferiorly until its intersection with the vertebral column	Overestimates the inferior limit
Baird et al 2009	Nine cadaveric specimens	-	Rhinion	Within 3 to 5 mm from the base of the surgical resection	-	Performed on normal cadavers
Aldana et al 2011	Six cadaver specimens	Superior nostril-hard palate line (SN-HP Line)	Superior aspect of the nostril	Anterior border of C1-C2 junction	-	Performed on normal cadavers, underestimates the inferior limit
Aldana et al 2012	Nine cadaver specimens	Naso-axial line (NAxL)	Midpoint of the distance from rhinion to the anterior nasal spine of the maxillary bone	Tip of the posterior nasal spine of palatine bone	Posteriorly and inferiorly until its intersection with the vertebral column	Performed on normal cadavers, overestimates the lower limits
La Corte et al 2015	Six patients (4 adult and 2 pediatric)	Rhinopalatine Line (RPL)	The two-thirds point of the distance from the rhinion to the anterior nasal spine of the maxillary bone	Posterior nasal spine of the palatine bone	Posteriorly and inferiorly until its intersection with the vertebral column	

* The most inferior point on the nasal bone

§ All lines were taken in the mid sagittal plane of a computed tomography (CT) scan

*Group 3: indications*: a variety of pathological lesions can affect the CCJ; ventral compression of this region can be produced by neoplasms, congenital disorders, infections, degenerative diseases and posttraumatic conditions [57]. The EEA to CCJ was first used by Kassam et al (2005) [9] to perform an odontoidectomy. Since then, the odontoidectomy is widely performed by the EEA as a treatment of congenital and acquired lesions causing compression of the ventral CCJ [13, 15, 18–21, 23, 24, 26, 35, 39, 44] including pediatric patients as well [14, 22, 30–32, 38, 40]. Most recent review of literature [58, 59] concluded that the rheumatoid pannus and basilar invagination were the most common indication of EEA to CCJ. Other case reports described the use of EEA approach for aneurysm clipping on the vertebral artery [10], for the excision of ventral posterior fossa meningioma [60] and for tuberculosis [61]. Recently, Mendez et al (2015) [62], by a cadaveric study, demonstrated the feasibility of C1-2 fusion via EEA, this novel technique could be the alternative of the second time C1-C2 posterior fixation.

*Group 4: biomechanical stability following the approach*: the CCJ moves in three directions, flexion-extension, axial rotation and lateral bending. Two cadaveric biomechanical studies [63, 64] from St. Joseph's Hospital, Phoenix, Arizona, USA concluded that the removal of the inferior-third of clivus associated with an intradural exposure to the ventral CCJ and foramen magnum led to hypermobility at Occiput-C1 junction. However, these studies stated that the craniocervical fusion is clinically indicated only after removal of 75% of anterior condyle. The EEA to CCJ provides the possibility to avoid the morbidity of the transoral approach by keeping the incision in the nasopharynx above the level of the soft palate; therefore, it affects early extubation and early feeding [60, 65]. The approach provides sufficient surgical field [54] and through it, selected cases of odontoidectomy can be performed with the preservation of C1 anterior arch, thus, it potentially reduces the instability of CCJ [18, 20, 66, 67]. Additional techniques to support the approach have been proposed. Some authors use the navigation [18, 24, 28, 29, 42, 68]; others offered a technique to preserve the mucosal integrity of the posterior nasal septum and pedicled nasoseptal flap [69]; the combination with the trans oral approach was also described [17, 70]. The main challenge of the EEA to CCJ is the presence of anatomical structures that can limit the surgical approach, including the bony and soft tissues of the nose superiorly and the hard palate inferiorly. Many studies aimed to determine the feasibility of the approach by using anatomical landmarks to determine the inferior limit and by studying the effect of head and neck position in widening the surgical field [12, 25, 46–56]

## Conclusion

This is the first paper reviewing all available clinical, anatomical and biomechanical studies on the EEA to CCJ. This unique approach went through significant steps of evolution since its first description in 2002. The EEA became the approach of choice to the ventral CCJ. It is significantly less invasive compared to the trans oral approach and carries less potential side effects. The accurate prediction of the inferior limitation is now potentially feasible. However, we still need a standard method to evaluate the limitations. The approach still needs a work out to be applied for more indications especially the fusion.

### What is known about this topic

- Two papers reviewed the available literature (clinical series) about the endoscopic endonasal approach to craniocervical junction.

### What this study adds

- This is the first paper that covers all the available literature including the cadaveric studies and the case reports as well.

## Competing interests

The authors declare no competing interest.
